# Effect of care bundles for acute kidney injury: A systematic review and meta-analysis

**DOI:** 10.1371/journal.pone.0302179

**Published:** 2024-04-17

**Authors:** Shuzhen Zhang, Yixin Chen, Fangfang Zhou, Lailiang Wang, Qun Luo

**Affiliations:** Department of Nephrology, Ningbo No.2 Hospital, Ningbo, Zhejiang Province, People’s Republic of China; Azienda Ospedaliero Universitaria Careggi, ITALY

## Abstract

**Purpose:**

Acute kidney injury (AKI) is frequent among in-hospital patients with high incidence and mortality. Implementing a series of evidence-based AKI care bundles may improve patient outcomes by reducing changeable standards of care. The aim of this meta-analysis was therefore to appraise the influences of AKI care bundles on patient outcomes.

**Materials and methods:**

We explored three international databases (PubMed, Embase, and Cochrane Central Register of Controlled Trials) and two Chinese databases (Wanfang Data and China National Knowledge Infrastructure) for studies from databases inception until November 30, 2022, comparing the impact of different AKI care bundles with usual standards of care in patients with or at risk for AKI. The study quality of non-randomized controlled trials and randomized controlled trials was evaluated by the NIH Study Quality Assessment Tool and the Cochrane risk of bias tool. Heterogeneity between studies was appraised by Cochran’s Q test and I^2^ statistics. The possible origins of heterogeneity between studies were assessed adopting Meta-regression and subgroup analyses. Funnel plot asymmetry and Egger regression and Begg correlation tests were performed to discover potential publication bias. Data analysis was completed by software (RevMan 5.3 and Stata 15.0). The primary outcome was short- or long-term mortality. The secondary outcomes involved the incidence and severity of AKI.

**Results:**

Sixteen studies containing 25,690 patients and 25,903 AKI episodes were included. In high-risk AKI patients determined by novel biomarkers, electronic alert or risk prediction score, the application of AKI care bundles significantly reduced the AKI incidence (OR, 0.71; 95% CI, 0.53–0.96; p = 0.02; I^2^ = 84%) and AKI severity (OR, 0.59; 95% CI, 0.39–0.89; p = 0.01; I^2^ = 65%). No strong evidence is available to prove that care bundles can significantly reduce mortality (OR, 1.16; 95% CI, 0.58–2.30; p = 0.68; I^2^ = 97%).

**Conclusions:**

The introduction of AKI care bundles in routine clinical practice can effectively improve the outcomes of patients with or at-risk of AKI. However, the accumulated evidence is limited and not strong enough to make definite conclusions.

## 1 Introduction

Acute kidney injury (AKI) is frequent among in-hospital patients with high incidence and mortality [1]. Owing to the absence of effective pharmacotherapies for AKI treatment, existing international guidelines focus on early recognition and timely intervention [2]. Advances in diagnostic tools, electronic alerts and novel renal biomarkers, such as tissue inhibitor of metalloproteinases-2 and urine insulin-like growth factor-binding protein 7, have enabled the early identification of AKI [3, 4]. However, early diagnosis does not improve patient outcomes by physician behavior, possibly because of great variations across routine clinical care. Care bundles, described as a set of simple evidence-based practices, have been proposed as tools to improve patient outcomes by reducing variability in standards of care [5]. The core elements of AKI bundles include the amelioration of hemodynamic status, avoidance of nephrotoxic agents, and prevention of hyperglycemia [6]. In several clinical situations, care bundles have been applied to patients with or at risk for AKI. Some isolated articles or reviews have reported patient-related benefits of care bundles including lower mortality and inhibition of AKI progression such as the original report by Kolhe et al. [7] and narrative review by Sykes et al. [8]. However, in reality, the overall care bundle compliance remains poor [7]. On the other hand, other studies report contrary findings. Joslin et al. found significant improvements in recognition, fluid assessment and nephrotoxic cessation following introduction of AKI bundle; however, these improvements were not correlated with enhanced patient outcomes [9].

The objectives of this study were to appraise the effects of AKI care bundles on patient outcomes, summarize the bundle components, and identify the key bundle care implementation strategies, which can improve compliance with AKI care bundles.

## 2 Methods

The study protocol followed the Preferred Reporting Items for Systematic Reviews and Meta-Analyses (PRISMA) guidelines [10, 11].

### 2.1 Search strategy

Two separate investigators (Shuzhen Zhang, Yixin Chen) searched the following electronic databases from database inception until November 30, 2022: PubMed, Embase, Cochrane Central, Wanfang Data, and China National Knowledge Infrastructure. Irrespective of the publication status or language, we searched for studies comparing the effects of AKI care bundles with usual standard of care in patients with or at risk for AKI. Our search strategy included the selection of key words and subject headings related to AKI and care bundles, including: “acute kidney injury”, “acute kidney failure”, “acute renal failure”, “patient care bundle”, “care checklist”, “prevention bundle”, etc. S1 Table shows the details of the search strategy. The references in the studies selected were further used to search for other related studies.

### 2.2 Inclusion and exclusion criteria

Inclusion criteria for this meta-analysis were: 1) in-hospital adult patients with or at high risk of AKI (identified using a laboratory or electronic alert system); 2) patients managed using AKI care bundles; 3) at least one AKI outcome (short- and long-term mortality, AKI incidence, or AKI severity); and 4) (quasi) randomized trials or observational studies. An AKI care bundle was a series of simple evidence-based diagnostic and therapeutic interventions collectively planned and performed in line with the Institute for Healthcare Improvement. The basic elements of the AKI bundle were set as advised by the guidelines of Improving Global Outcomes (KDIGO) or other relevant AKI guidelines [12].

According to the recommendations of the KDIGO Guideline, studies were excluded if:1) they were studies from letters/editorials, conference abstracts, comments, or reviews; 2) studies without sufficient information for analysis; and 3) there were no measured outcomes.

### 2.3 Study selection

Two investigators independently filtered all titles and abstracts to select pertinent articles, retrieved the full-text copies of the relevant studies for closer inspection to determine study eligibility and resolved any disagreement by consulting with a third investigator.

**Fig 1:**
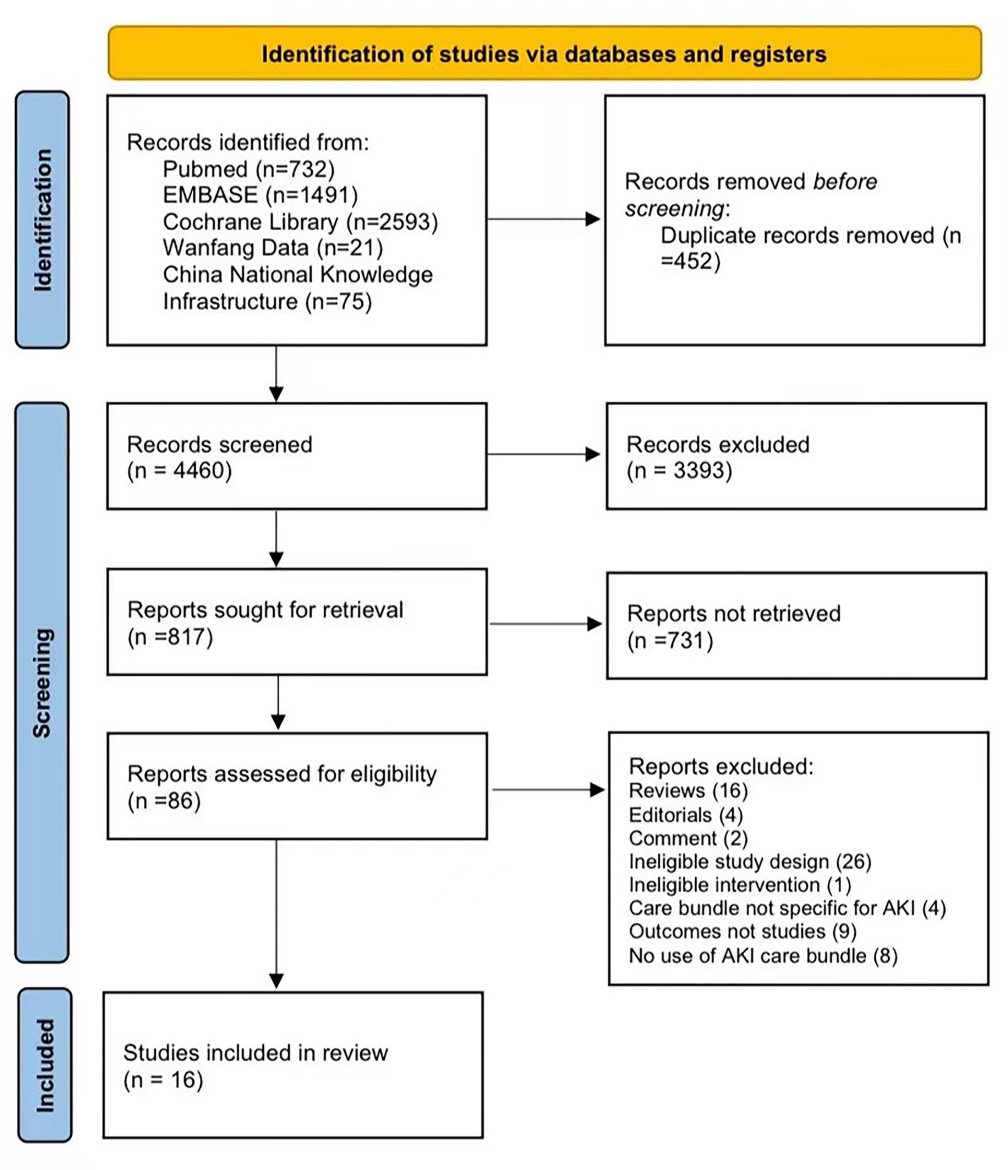
PRISMA 2020 flow diagram of study selection.

Initially, 4912 citations were identified. 86 potentially pertinent full-text studies were read for further appraisal after reduplication and filtering all titles and abstracts. Finally, 16 studies were involved in this meta-analysis. See the PRISMA diagram for the detailed procedure **(Fig 1**).

### 2.4 Data extraction

Study characteristics (i.e., year, design, setting, and country), the number of participants, the intervention, comparison groups, and the outcomes were retrieved from individual studies by two investigators and summarized in evidence tables. The characteristics of the AKI bundles were also summarized.

Implementation strategies included in care bundles were categorized by referring to the taxonomy developed by the Cochrane EPOC [13].

When studies that met the inclusion criteria lacked significant primary data, we attempted to contact the authors.

### 2.5 Quality assessment and data synthesis

The bias risk in cohort studies was appraised by the Newcastle-Ottawa scale [14]. This evaluation scale rates the quality of the included studies pursuant to three groups: the selection of the study groups, the comparability of the groups, and the ascertainment of the study outcomes. Scores of 7–9, 4–6, and <4 were classified as having a low, moderate, or high risk of bias, respectively. We also used the ROBINS-I to evaluate the risk of bias in cohort studies, which address key criteria such as selection bias, exposure measurement, blinding, completeness of outcome data and selectivity of reporting [15]. Bias risk in randomized controlled trials (RCTs) was appraised by Cochrane Risk of Bias Assessment Tool [16], review authors’ judgements about each risk of bias item for each included study. To identify any potential publication bias, both statistically and visually, Egger regression and Begg correlation tests and funnel plot asymmetry were conducted.

This meta-analysis was completed utilizing the Cochrane Collaboration Review Manager (RevMan 5.3) and the Cochran–Mantel–Haenszel analysis (odds ratio [OR] reported with the 95% confidence interval [CI]). Data were combined using a random effects model, given the expected diversity of clinical approaches and methodologies used in the included studies. Cochran’s Q test and I^2^ test statistics were applied to evaluate the heterogeneity between studies [17] and analyzed using STATA version 15.0 software (STATA Corp, College Station, Texas). I^2^ index less than 25% indicates low heterogeneity, between 25% and 75% indicates moderate heterogeneity, and more than 75% indicates high heterogeneity. Due to heterogeneity in the studies, a random effects model was used to perform meta-analysis. Interactions between subgroups were tested by Meta-regression models, and a p value ≤ 0.05 was considered as statistical significance. Six trial features (study design, country, care bundle compliance, setting, care bundle components and implementation strategies) were defined as covariates. Univariate meta-regression was used to assess the association between each covariate and each outcome (short- or long-term mortality, AKI incidence and AKI severity). Subgroup analyses were carried out according to the source of heterogeneity.

## 3 Results

### 3.1 Study characteristics

**Table 1** recapitulates the baseline characteristics of the included studies. Four of the involved studies were RCTs [4, 18–20], 8 were before-after studies [9, 21–27], and the remaining 4 were prospective observational studies [6, 7, 28, 29].

**Table 1:** Baseline characteristics of the included studies.

Author and year (Reference)	Country	Total No. of Patients (intervention- control /after-before)	Care bundle compliance (%)	Design	Setting	Care bundle and implementation strategies	Outcomes
Tsui 2014 et al. [26]	UK	53/55	100	Uncontrolled before-after study.	ED	11 elements (baseline creatinine, fluid status, urinalysis, med review × 2, u PCR, urine output, USS, referral × 3) Education and audit feedback.	1.Median length of stay; 2.Admission to HDU; 3.Admission to ITU; 4.Mortality (%).
Joslin 2015 et al. [9]	UK	92/100	8.4	Uncontrolled before-after study.	ED	8 elements (Assessment, fluid therapy, hyperkalaemia, urinalysis, medication review, creatinine monitor, USS, fluid documentation) Education and audit feedback.	Hospital mortality
Kolhe 2015 et al. [7]	UK	306/2194*	21.6	Prospective observational study.	The whole hospital	6 elements’ AUDITS’ (fluid assessment, urinalysis, diagnose cause of AKI, order investigations, initiate treatment, refer) Education.	1.In-hospital case fatality; 2.LOS; 3.Progression of AKI stages.
Kolhe 2016 et al. [24]	UK	939/1823	25.6	Uncontrolled before-after study.	The whole hospital	6 elements’ AUDITS’ (fluid assessment, urinalysis, diagnose cause of AKI, order investigations, initiate treatment, refer) Education.	Primary outcome: All-cause in-hospital case–fatality. Secondary outcomes: 1.Progression of AKI to higher stages; 2.LOS; 3.Survival post-discharge.
Mayne 2016 et al. [25]	France	107/104	Not stated	Uncontrolled before-after study.	Surgery	5 elements ABCDE checklist (Address medications, Boost blood pressure, Calculate fluid balance, Dipstick of urine, Exclude obstruction) Education.	AKI incidence
Meersch 2017 et al. [20]	Germany	138 /138	100	RCT	Cardiac surgery	KDIGO bundle: optimization of volume status and hemodynamics, avoidance of nephrotoxic drugs, and preventing hyperglycemia, close monitoring of serum creatinine and urine output, consideration of alternatives to radiocontrast agents. Unclear.	Primary outcome: The rate of AKI within the first 72 h. Secondary outcome: 1.AKI severity,; 2.Need for dialysis; 3.LOS; 4.MAKE at days 30, 60, and 90.
Gocze 2018 et al. [18]	Germany	60/61	Not stated	RCT	ICU	KDIGO bundle: optimization of fluid status, maintenance of perfusion pressure, discontinuation of nephrotoxic agents. Unclear.	Primary outcome: Overall AKI. Secondary outcomes: AKI stage II and III.
Hodgson 2018 et al. [22]	UK	7881/6862	85	Uncontrolled before-after study.	ED	8 elements (cause, fluid, drug, urinalysis, escalation of care, USS, other specific blood tests, nephrogical consultation) Education, multidisciplinary team.	Primary outcome: Change in incident AKI. Secondary outcomes: 1.In-hospital mortality; 2.AKI progression; 3.Escalation of care.
Schanz 2019 et al. [4]	Germany	54/46	100	RCT	ED	KDIGO bundle: optimization of haemodynamics and fluid status, avoidance/stopping of nephrotoxic drugs, preventing hyperglycaemia Nephrogical consultation.	Primary outcome: The incidence of moderate to severe AKI within the first day after admission. Secondary outcomes: 1.AKI occurrence within 3days after admission; 2.Need for RRT; 3.Length of hospital stay and death.
Wang 2019 et al. [27]	China	136/132	91.1	Uncontrolled before-after study.	ICU	6 elements (fluid status, fluid assessment, fluid resuscitation guides by CVP, maintaining a MAP of at least 65mmHg, adjust the use of antibacterial drugs according to creatinine clearance, CRRT, avoidance/stopping of nephrotoxic drugs) Unclear.	1.Infused fluid on the first day and third day; 2.The ratio of lung protective ventilation and the non-mechanical ventilation time; 3.Length stay in ICU and kidney replacement rate.
Pan 2019 et al. [29]	China	103/68	100	Prospective observational study.	Cardiac surgery	4 elements (volume and hemodynamic status were optimized depending on PiCCO parameters to a goal of cardiac index (CI) > 41.68 mL·s^-1^·m^-2^, global end diastolic volume index (GEDVI) > 700 mL/m^2^ or intrathoracic blood volume index (ITBVI) > 850 mL/m^2^, extravascular lung water index (EVLWI) < 10 mL/kg, and MAP > 65 mmHg) Unclear.	1.CVP within 24 hours. 2.MAP at 8 hours and 16 hours. 3.SOFA score at 24 hours. 4.Dominant liquid equilibrium quantity at 24 hours, 48 hours and 72 hours. 5.Incidence of combination with AKI during 72 hours. 6.Incidence of moderate to severe AKI. 7.Usage of CRRT. 8.Duration of mechanical ventilation. 9.The length of ICU stay. 10.The post-operation complications (except AKI). 10.7-day and 28-day mortality.
Abdelaziz 2020 et al. [6]	Egypt	306/2194	Not stated	Prospective observational study.	ICU	5 elements (Early recognition of transient oliguria, minimization of nephrotoxic medications, stabilization of hemodynamic status, judicious use of blood transfusion and nephrology consultation) Nephrogical consultation.	Primary outcome: The incidence of AKI among critical care patients within 48 h of ICU admission. Secondary outcomes: All secondary outcomes are measured within 30 days. 1.30 days all-cause mortality; 2.Time till recovery after development of AKI.
Engleman 2020 et al. 21]	USA	338/338	Not stated	Uncontrolled before-after study.	Cardiac surgery	KDIGO bundle: avoidance of nephrotoxic agents, discontinuation of ACEi/ARB, avoidance of hyperglycemia, avoidance of radiocontrast agents, close monitoring of urinary output, goal-directed fluid therapy. Multidisciplinary team.	Primary outcome: The development of stage 2 or 3 AKI. Secondary outcome: 1.The development of stage 1 AKI; 2.Length of stay; 3.Cost of hospitalization; 4.Perioperative mortality and readmission rate.
Koeze 2020 et al. [23]	Netherlands	1347/1295	Not stated	Uncontrolled before-after study.	ICU	5 elements (optimizing the fluid balance, discontinuation of diuretics, maintaining a MAP of at least 65mmHg, critical evaluation of the indication and dose of nephrotoxic drugs) Education.	Primary outcome: 1.Mortality; 2. RRT; 3.Progression of AKI. Secondary outcomes: 1.The severity of AKI; 2.ICU length of stay; 3.In-hospital mortality.
Zarbock 2021 et al. [19]	Germany	136/142	65.4	RCT	Cardiac surgery	4 elements (optimization of volume status and hemodynamics, functional hemodynamic monitoring, avoidance of nephrotoxic drugs, prevention of hyperglycemia) Unclear.	Primary outcome: The compliance rate to the KDIGO bundle. Secondary outcomes: 1.The occurrence and severity of AKI within 72 hours; 2.Renal recovery at days 30, 60, and 90; 3.All-cause mortality at days 30, 60, and 90; 4.Length of stay in ICU and hospital; 5.Use of RRT at days 30, 60, and 90; 6. MAKE at day 90.
Kotwal 2022 et al. [28]	Australia	265/374	94.8	Prospective observational study	The whole hospital	4 elements (early identification, an interruptive eAlert, a management guide, an education programme) Nephrogical consultation.	Primary outcomes: LOS and all-cause in-hospital mortality. Secondary outcomes: aspects of AKI management quality.

*2500 episodes and 2297 patients. No. = number; UK = the United Kingdom; USA = the United States of America; ED = Emergency Department; ICU = Intensive Care Unit; AKI = acute kidney injury; u PCR = urinary protein:creatinine ratio; USS = ultrasound scan; CVP = central venous pressure; MAP = mean arterial pressure; RRT = renal replacement therapy; CRRT = continuous renal replacement therapy; ACEi = angiotensin converting enzyme inhibitors; ARB = angiotensin II receptor blockers; PiCCO = pulse indicator continuous cardiac output; SOFA = sequential organ failure assessment; HDU = high dependency unit; ITU = intensive care unit; RCT = random controlled trial; MAKE = major adverse kidney events; LOS = length of stay

All included studies analysed the effectiveness of care bundles within in-patient settings. Three studies included in-hospital patients [7, 24, 28], four studies included only emergency department patients [4, 9, 22, 26], four studies assessed ICU patients [6, 18, 23, 27], and the remaining studies included patients who underwent surgery [19–21, 25, 29].

All of the included studies were performed between 2014 and 2022; 11 of those were conducted in Europe [4, 7, 9, 18–20, 22–26], two in China [27, 29], one in the United States [21], one in Australia [28], and one in Egypt [6].

The care bundle components chosen for the intervention greatly differed among studies, both quantitatively (ranging from 4 to 11components) [6, 7, 9, 19, 22–29] and qualitatively(four studies were based on the KDIGO Guideline) [4, 18, 20, 21]. These components included the improvement of fluid and hemodynamics status, avoidance or stopping of nephrotoxic drugs, and prevention of hyperglycemia.

Two studies [9, 26] used audit and feedback, and five studies [4, 6, 21, 22, 28] used multidisciplinary teams or nephrological consultation as strategies for implementation of the care bundle.

### 3.2 Quality assessment

**Fig 2:**
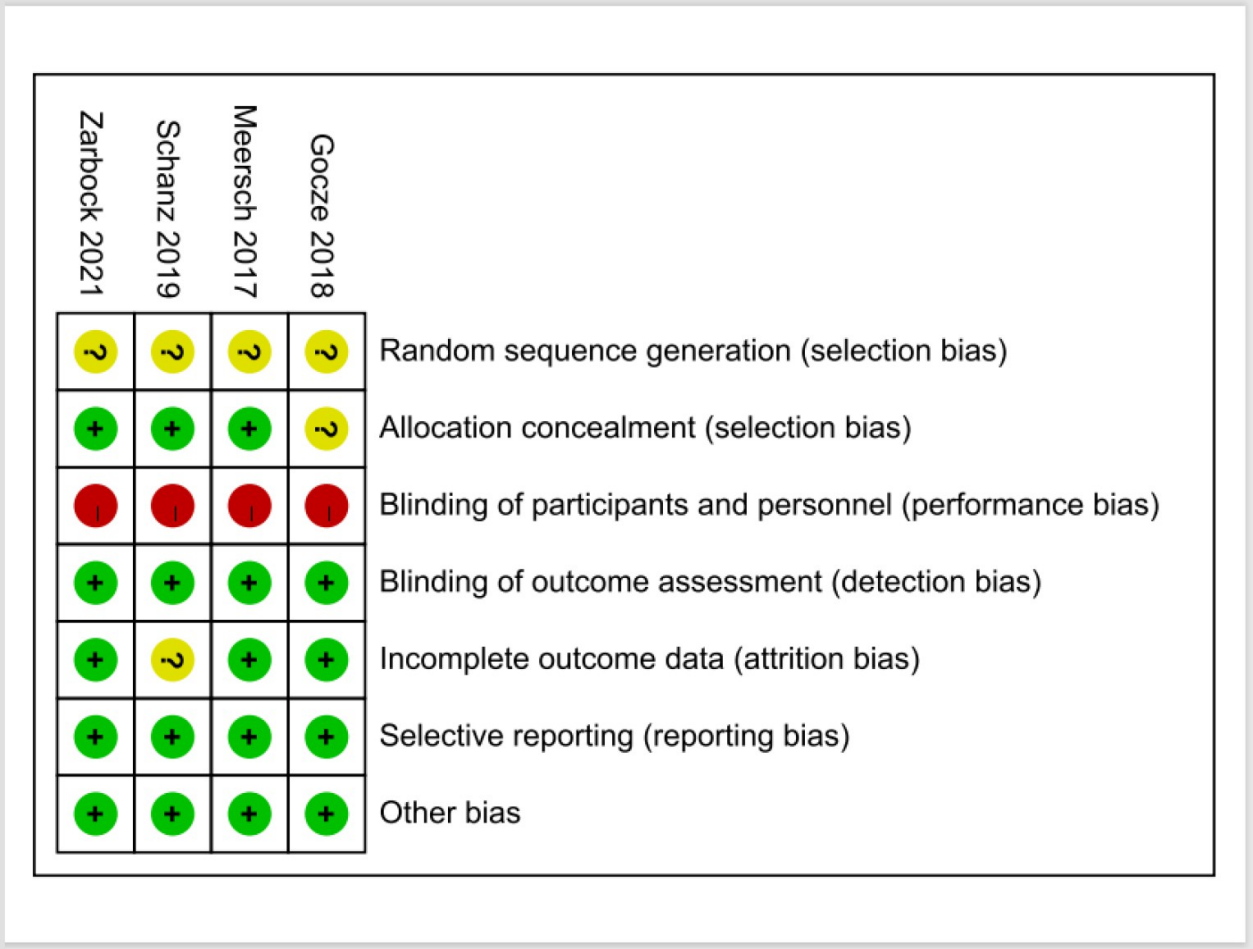
The Cochrane Risk of Bias Assessment Tool was used to assess risk of bias for the randomized, controlled trials. Red, high risk of bias; Green, low risk of bias; Yellow, uncertain risk of bias.

The bias risks of cohort studies involved in this study low -to- moderate risk. The quality of the involved studies was appraised by the Newcastle–Ottawa Scale (**Table 2**). We also used the ROBINS-I to evaluate the risk of bias in cohort studies, the detailed results are available in **Table 3**. Four RCTs were involved in the analysis (**Fig 2**) and assessed using the Cochrane Risk of Bias Assessment Tool.

**Table 2:** The Newcastle–Ottawa Scales were used to assess risk of bias for the cohort studies. Each domain was rated on a scale of zero or one star, except comparability, which can be awarded up to two stars. 0, High or unclear risk of bias; 1 or 2, low risk of bias (scores ≥ 7–9, 4–6, <4 are considered low, intermediate, and high risk, respectively).

Author and Year (Reference)	Representative Cohort	Selection of Nonexposed	Exposure Ascertained	Outcome not present at outset	Comparability	Outcome Assessment	Duration of Follow-Up	Adequacy of Follow-Up	Overall
Tsui 2014 et al. [26]	1	1	1	1	2	1	0	1	8/9
Joslin 2015 et al. [9]	1	1	1	1	2	1	0	1	8/9
Kolhe 2015 et al. [7]	1	1	1	1	2	1	0	1	8/9
Kolhe 2016 et al. [24]	1	1	1	1	2	1	0	1	8/9
Mayne 2016 et al. [25]	0	1	1	1	2	1	1	1	8/9
Hodgson 2018 et al. [22]	1	1	1	1	2	1	0	1	8/9
Wang 2019 et al. [27]	0	1	1	1	2	1	0	0	6/9
Pan 2019 et al. [29]	0	1	1	1	2	1	0	1	7/9
Abdelaziz 2020 et al. [6]	1	1	1	1	2	1	0	1	8/9
Engleman 2020 et al. [21]	0	1	1	1	2	1	1	1	8/9
Koeze 2020 et al. [23]	1	1	1	1	2	1	0	1	8/9
Kotwal 2022 et al. [28]	0	1	1	1	2	1	1	1	8/9

**Table 3:** The ROBINS-I were used to assess risk of bias for the cohort studies.

Author and Year (Reference)	Bias due to confounding	Bias in selection of participants into study	Bias in classification of interventions	Bias due to deviations from intended intervention	Bias due to missing data	Bias in measurement of outcomes	Bias in selection of the reported result	Overall risk of bias
Tsui 2014 et al. [26]	NA*	Low	Low	Low	Low	Moderate	Low	Low
Joslin 2015 et al. [9]	NA*	Low	Low	Low	Low	Moderate	Low	Low
Kolhe 2015 et al. [7]	NA*	Low	Low	Low	Low	Moderate	Low	Low
Kolhe 2016 et al. [24]	Low	Low	Low	Low	Moderate	Moderate	Low	Low
Mayne 2016 et al. [25]	NA*	Low	Low	Low	Low	Moderate	Low	Low
Hodgson 2018 et al. [22]	NA*	Low	Low	Low	Low	Moderate	Low	Low
Wang 2019 et al. [27]	NA*	Low	Low	Low	Low	Moderate	Low	Low
Pan 2019 et al. [29]	Low	Serious	Moderate	Low	Low	Moderate	Low	Moderate
Abdelaziz 2020 et al. [6]	Moderate	Low	Low	Low	Low	Moderate	Low	Low
Engleman 2020 et al. [21]	NA*	Low	Low	Low	Low	Moderate	Low	Low
Koeze 2020 et al. [23]	NA*	Low	Low	Low	Low	Moderate	Low	Low
Kotwal 2022 et al. [28]	Moderate	Low	Low	Low	Low	Moderate	Low	Low

*Confounding domain not applicable to before-after studies

### 3.3 AKI incidence

**Fig 3:**
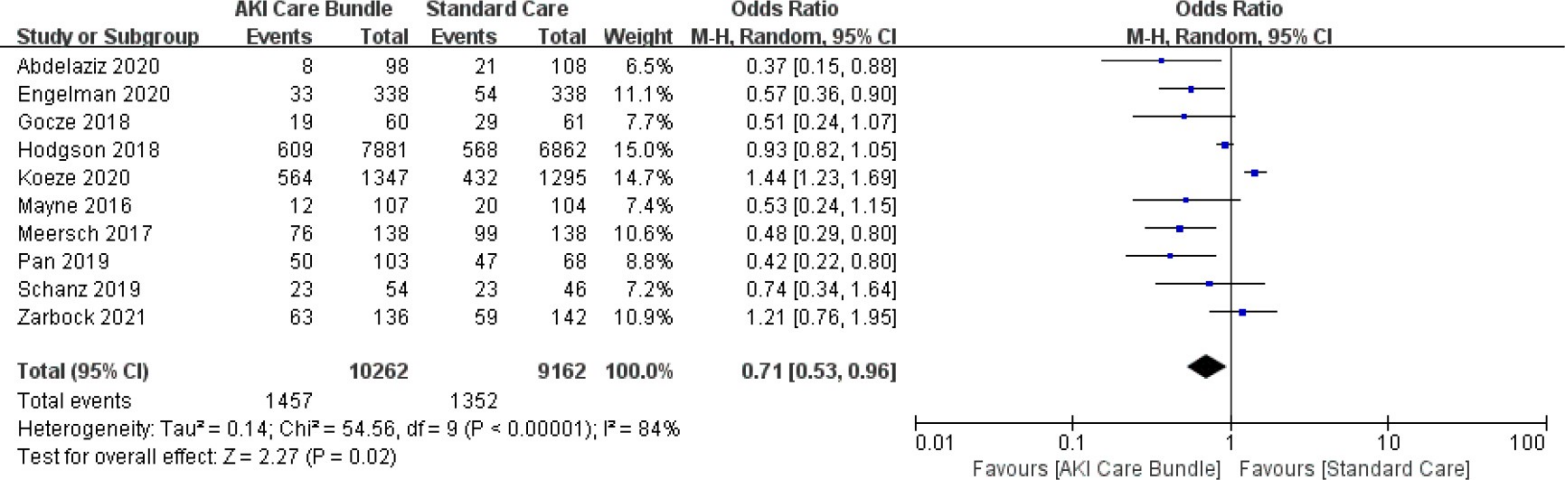
AKI incidence with and without AKI care bundles.

**Fig 4:**
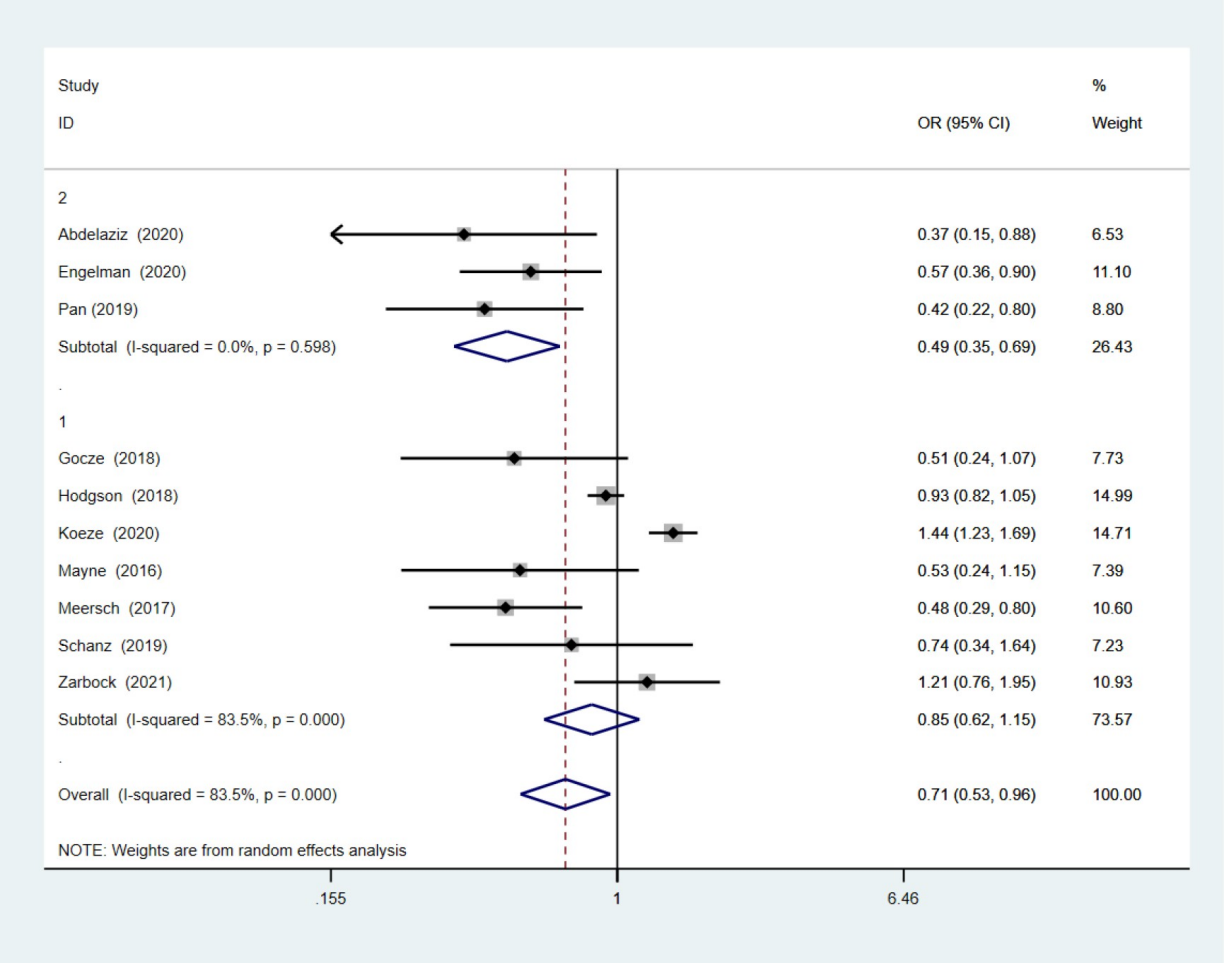
Sub-analysis of both European countries and other countries.

Ten studies evaluated changes in the AKI incidence following implementation of the care bundles [4, 6, 18–23, 25, 29] in 19,424 patients. Despite the high inconsistency in the study findings [Q (9) = 54.56; p < 0.00001; I^2^ = 84%], in the random effects method results, most studies showed a significant decrease in the AKI incidence, the overall OR was 0.71 (0.53–0.96; p = 0.02) (**Fig 3**). The midpoint of each line and the length of the line respectively show the mean difference and its 95% confidence interval for each study. The size of squares represent the weight which that study had on the overall summary effect. The middle of the diamond sign shows the summary effect and the horizontal diameter of it represent 95% confidence intervals of the summary effect. We conducted a sub‐analysis of studies from European countries (subgroup 1) and other countries (subgroup 2) (**Fig 4**). The studies from European countries continued to demonstrate high heterogeneity with an I^2^ of 83.5%; however, the heterogeneity was markedly decreased for studies from other countries, with an I^2^ of 0. Studies from other countries (OR = 0.487; 95%CI, 0.345–0.688; p < 0.001) continued to report decreased AKI occurrence. Studies from European countries (OR = 0.847; 95%CI, 0.621–1.155; p = 0.293) showed a non-significant association between AKI incidence and implementation of the AKI care bundles. Meta-regression analysis showed there were no statistically significant associations (p > 0.05) between AKI incidence and any of the following intervention characteristics: care bundle compliance, study design, setting, care bundle components and implementation strategies.

**Fig 5:**
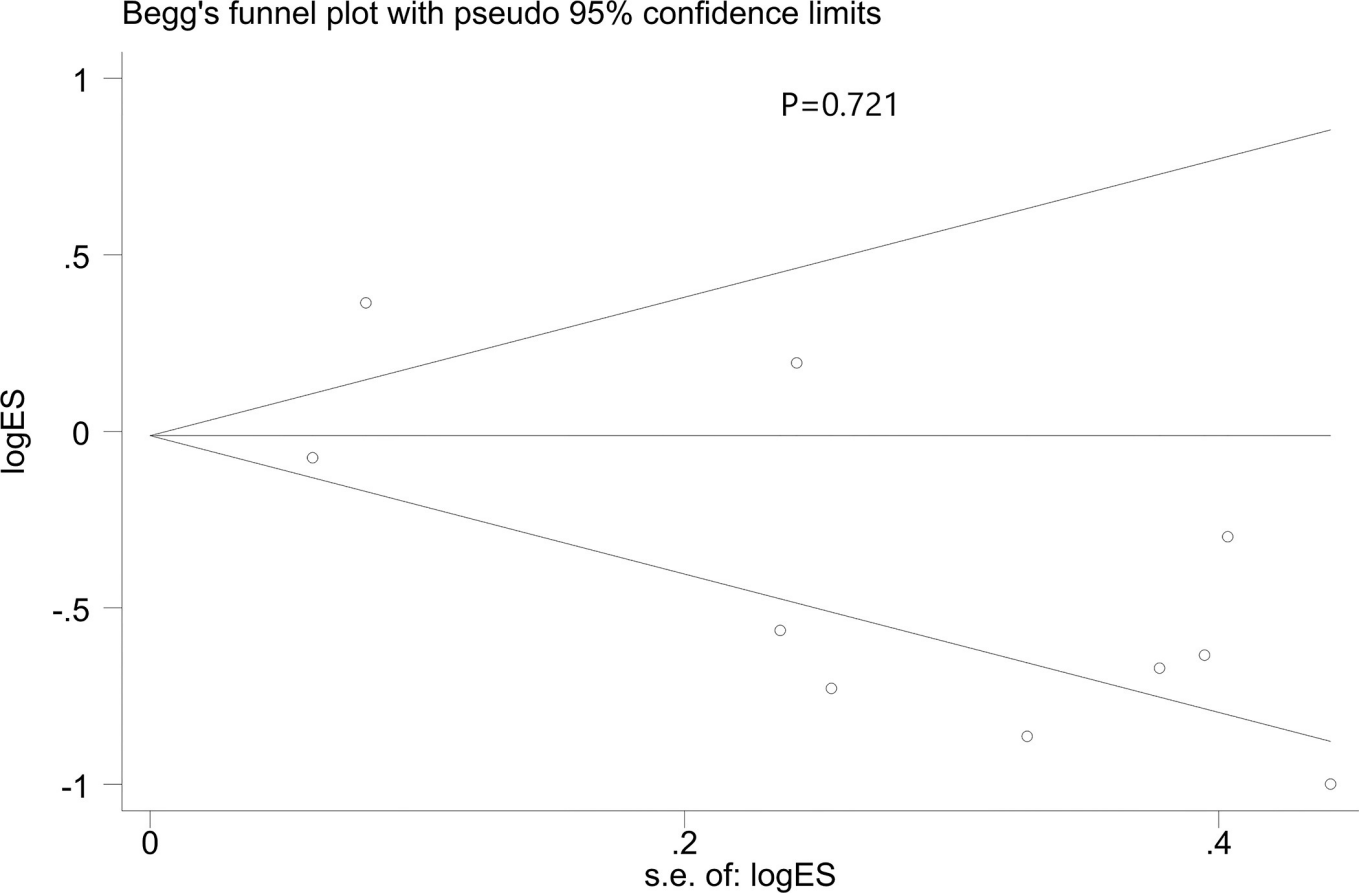
Begg’s funnel plot of AKI incidence after using AKI care bundles. s.e. = standard error.

No publication bias was discovered by statistical tests such as Egger regression (p = 0.053) and Begg correlation (p = 0.721) tests. Although the funnel plot demonstrated asymmetry (**Fig 5**).

### 3.4 AKI severity

**Fig 6:**
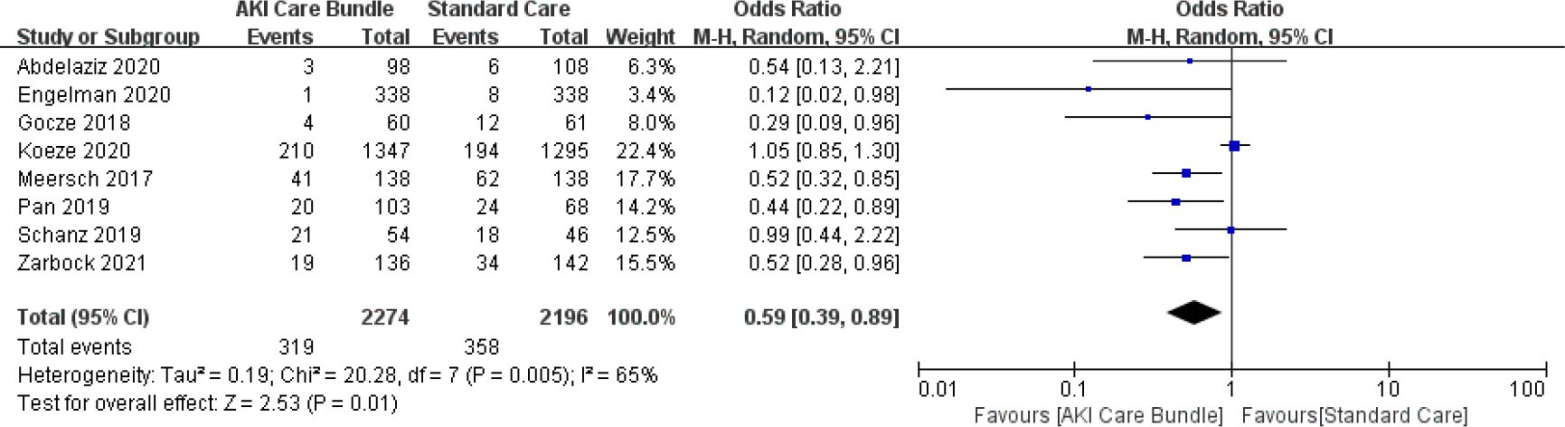
AKI severity with and without AKI care bundles.

**Fig 7:**
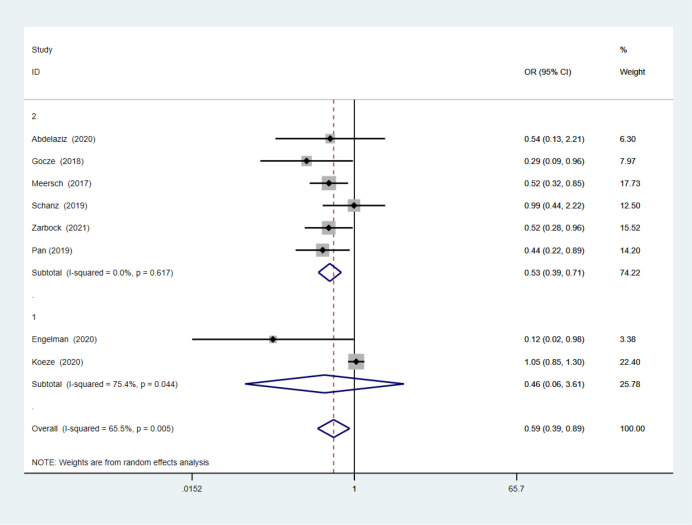
Sub-analysis of both uncontrolled before-after studies and prospective observational and RCTs.

A pooled analysis of 8 studies (including 4,479 patients) [4, 6, 18–21, 23, 29] revealed that based on the Q and I^2^ index, there was moderate heterogeneity in the studies [Q (7) = 20.28; p = 0.005; I^2^ = 65%], so in the random effects method results, implementation of a care bundle bring on a significant decreases in moderate severe AKI occurrence (p = 0.01). The overall OR was 0.59 with 95%CI (0.39–0.89) (**Fig 6**). Meta-regression analysis showed there were no statistically significant associations (p > 0.05) between AKI severity and any of the following intervention characteristics: country, care bundle compliance, setting, care bundle components and implementation strategies. The sub–analysis aimed to distinguish uncontrolled before-after studies (subgroup 1) from prospective observational studies and RCTs (subgroup 2) (**Fig 7**). These results indicated that the uncontrolled before-after studies continued to demonstrate high heterogeneity with an I^2^ of 75.4%; however, heterogeneity was markedly decreased for the prospective observational studies and RCTs, with an I^2^ of 0.

Publication bias could not be appraised due to the number of included studies is small.

### 3.5 Short- and long-term mortality

**Fig 8:**
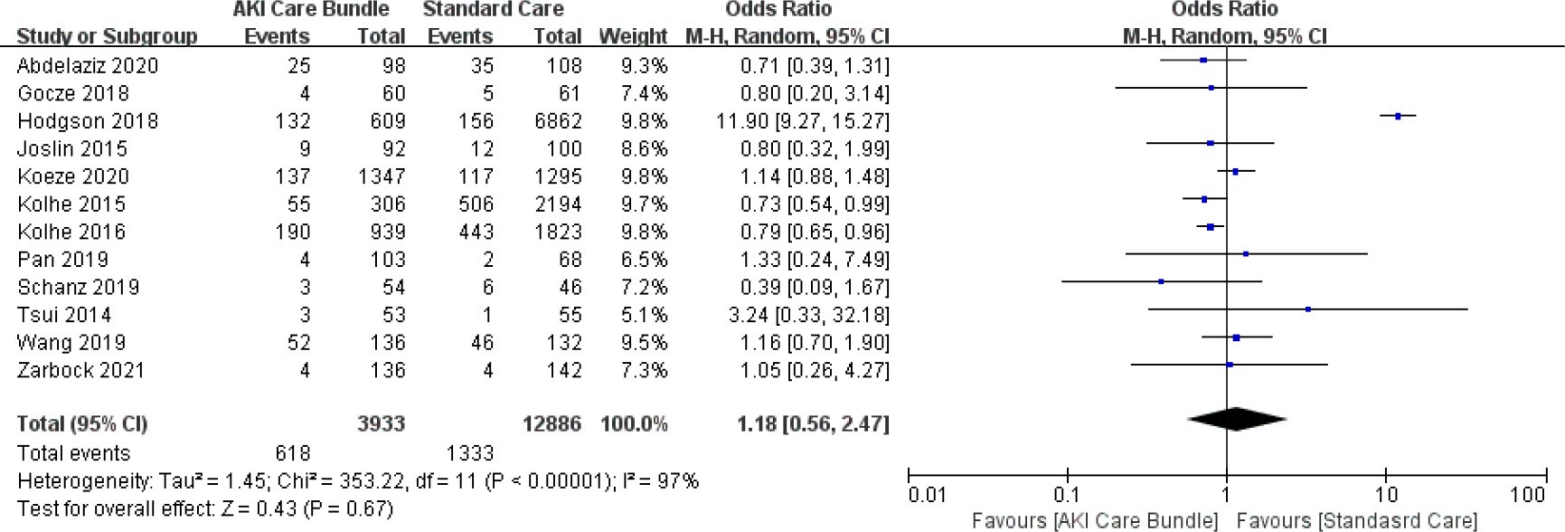
Short- and long-term mortality with and without AKI care bundles.

**Fig 9:**
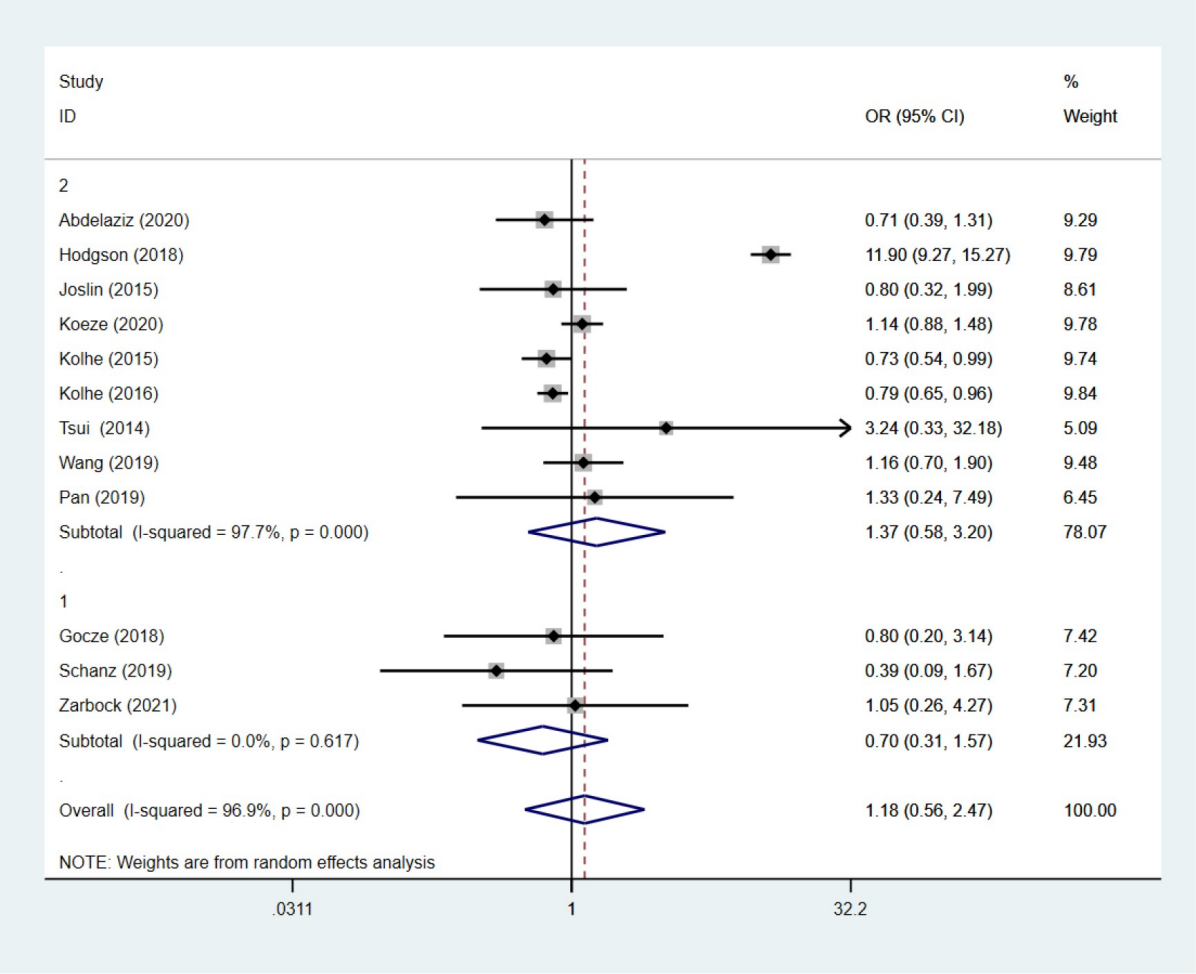
Sub-analysis of both studies with randomized controlled trials and non-randomized controlled.

Thirteen studies assessed changes in mortality after implementation of the AKI care bundles [4, 6, 7, 9, 18, 19, 22–24, 26–29] in 17,458 patients. There was a non-significant decrease in mortality (p = 0.68) with high heterogeneity between the results [Q (12) = 355.63; p = 0.68; I^2^ = 97%]. The overall OR was 1.16 with 95%CI (0.58–2.30) (**Fig 8**). Given the high heterogeneity of the results, we performed subgroup analysis (**Fig 9**). We divided the studies into subgroups 1 and 2. Subgroup 1 comprised RCTs, whereas subgroup 2 comprised non-RCTs. Non-RCTs continued to demonstrate high heterogeneity with an I^2^ of 97.4%; however, the heterogeneity was markedly decreased for RCTs (I^2^ = 0). Meta-regression analysis showed there were no statistically significant associations (p > 0.05) between short- and long-term mortality and any of the following intervention characteristics: country, care bundle compliance, setting, care bundle components and implementation strategies.

**Fig 10:**
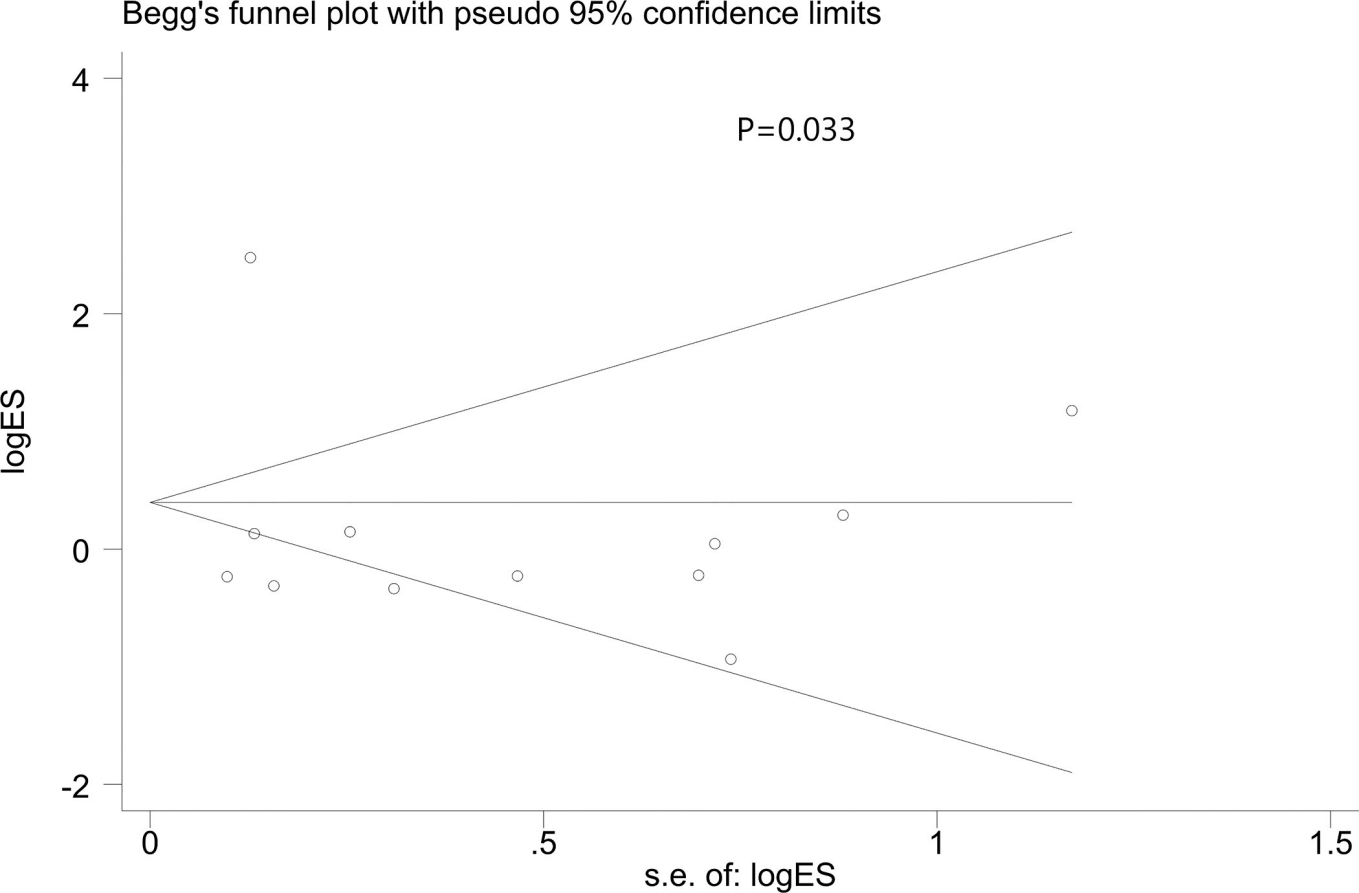
Begg’s funnel plot of Short- and long-term mortality after using AKI care bundles. s.e. = standard error.

Statistical tests Begg regression (p = 0.033) showed publication bias, and the funnel plot showed asymmetry (**Fig 10**). Although publication bias was not discovered by Egger correlation (p = 0.712) tests.

## 4 Discussion

We implemented a systematic search of the literature and recognized 16 studies involving 25,690 patients and 25,903 AKI episodes that assessed whether care bundles were valid in improving the outcomes of patients with or at-risk of AKI. This meta-analysis did not reveal any strong evidence that could prove that AKI care bundles significantly reduce mortality in all patients. Patients exposed to the AKI care bundles had a significantly reduced AKI incidence as compared with the usual standards of care. Moreover, a lessened incidence of moderate-to-severe AKI was noted in high-risk AKI patients.

### 4.1 AKI incidence

Compared to the general incidence of AKI, the utilization of an AKI care bundle in patients was associated with a lower incidence of AKI. Studies conducted in Egypt, the USA, and China showed decreases in AKI occurrence with low heterogeneity [6, 21, 29]. However, Meersch et al. failed to demonstrate any benefit from implementing care bundle in RCTs (OR = 0.70; 95%CI, 0.43–1.15; P = 0.16) [4, 18–20]. This reduction in terms of patient outcome could not be substantiated using randomized data due to inadequate sample sizes and variability in studied outcomes.

Each individual should receive optimal kidney-sparing treatment from a nephrological perspective [2]. The early detection of serum creatinine to diagnose AKI in high-risk patients is generally considered insufficient; furthermore, newer diagnostic tools, such as electronic alerts (E-alerts), stress/damage biomarkers, and risk stratification, have not yet been explored [30].

In recent years, E-alerts have garnered significant attention [31], as they can indicate early or impending AKI episodes and prompt rapid prevention measures along with early clinical evaluation and treatment strategies [32]. Regarding biomarkers, Schanz et al. proved that urinary tissue inhibitor of metalloproteinases-2(TIMP-2) and insulin-like growth factor-blinding protein 7 (IGFBP7) are beneficial in screening high-risk populations [4].

### 4.2 AKI severity

Previous studies have demonstrated a significant reduction in moderate-to-severe AKI following the implementing of a care bundle. However, uncontrolled before-after studies conducted by Schanz et al. failed to show any benefit from implementing the care bundle [4, 6, 23], perhaps attributable to insufficient statistical power to detect differences in AKI progression.

Several technology-based interventions have been investigated to improve AKI outcomes, including the development of a working conceptual model for studying the kinetics of AKI reversal and renal recovery [33]. However, a recent cluster-randomized trial [34] found no improvement in clinical outcomes when hospitals were randomized to introduce an alerting system. This could be due to the lack of the associated authoritative guidelines, which results in the lack of preventive or treatment measures in hospitals, and the lack of targeted education.

Care bundles can be used to improve care and make services more uniform. Substantial evidence supports the use of care bundles in hospital settings, such as emergency departments, wherein they have greatly improved the outcomes of patients with sepsis and community acquired pneumonia [35, 36]. AKI care bundles enable non-nephrologists to take action rapidly to prevent and treat AKI [35]. Therefore, early recognized AKI can be reversed by ensuring that the patients receive adequate fluid and medication to avoid the worsening of kidney function or any chance of toxicity.

### 4.3 Short- and long-term mortality in AKI

The implementation of AKI care bundles did not result in a significant reduction in short- or long-term mortality based on both randomized controlled trials (RCTs) and non-RCTs; however, the number of involved studies is small, thus the interpretation of findings should be cautiously done.

Selecting the appropriate patient may be crucial in successfully implementing an AKI care bundle. According to the studies by Hodgson et al. [22] and Koeze et al. [23], both studies involved in this review demonstrated a significant reduction in mortality following the implementation of an AKI care bundle. However, it should be noted that these studies enrolled large numbers of patients, which may have resulted in inaccurate outcomes.

Generally, inpatients are at a higher risk of developing AKI, which is associated with high mortality [1, 37, 38]. Consensus guidelines for AKI have recommended prompt treatment, including the maintenance of perfusion pressure, improvement of fluid status, avoidance of nephrotoxins, and prevention of hyperglycemia [2, 12]. Since AKI can be induced by hypovolemia and hypervolemia, optimization of fluid status is crucial [39]. The target blood pressure should be controlled at a mean arterial pressure >60–65 mmHg using vasopressors [12, 40]. In high-risk patients for AKI, the doses of nephrotoxic drugs and other drugs should be strictly controlled [12]. Recently, an observational and retrospective study [41] analyzed 5 interventions of the nephrology team with the potential to meliorate AKI outcomes, including the fluid adjustment and nephrotoxic withdrawal, etc. It reported that only fluid management lowered risk of starting renal replacement therapy (RRT) and progression to AKI stage 3, and none of the interventions reduced the risk of death in AKI patients. Hence, in the absence of evidence to endorse the application of other interventions to reduce AKI mortality. It suggested that multidisciplinary approach was demanded due to the multifactorial nature of AKI in hospitalized patients.

### 4.4 Strengths and limitations

Limited systematic studies have been conducted to evaluate the effectiveness of AKI care bundles; prior our study, only Schaubroeck et al. [42] presented a comprehensive review and meta-analysis on patient outcomes associated with AKI care bundle implementation. In particular, we extensively searched Chinese databases in order to include similar studies that could potentially serve as professional guidelines for Chinese hospitals. Our critical assessment of existing studies on AKI care bundles can inform further research planning, while our interpretation of the study results can aid in evaluating the local implementation and generalizability of AKI care bundles.

The effectiveness of this study is affected by the limited number of randomized studies, the small sample numbers in some of the individual studies and the wide differences in endpoints of studies. Furthermore, there were disparities in the quality of clinical care for AKI among included studies, with poorer quality associated with worse outcomes. Given that AKI often occurs under the management of healthcare professionals who are not nephrologists, it is crucial to enhance awareness about this condition among all healthcare professionals. Most studies applied a before-after design which has inherent limitations such as susceptibility to long-term trends bias and potential overestimation of the true effects attributed to AKI care bundles [43, 44].

## 5 Conclusion

Taken together, implementation of AKI care bundles in conventional clinical practice can effectively improve the prognosis of patients with or at risk of AKI. However, the accumulated evidence is limited and not robust enough to make definable conclusions.

## Supporting information

S1 Checklist PRISMA 2020 checklist. (DOCX)

S1 Table The details of the search strategy. (DOCX)
