# Supplementary material for: Effect of care bundles for acute kidney injury: A systematic review and meta-analysis
Source: PLoS One. 2024 Apr 17;19(4):e0302179. doi: 10.1371/journal.pone.0302179 (PMC11023357; doi:10.1371/journal.pone.0302179)
Supplement: S1 Table — (DOCX) [file pone.0302179.s002.docx]

# Supplementary Table 1

| Pubmed | #1. exp acute kidney injury/ |
| --- | --- |
|  | #2. (acute kidney injur*OR acute renal injur*).tw |
|  | #3. (acute kidney failure OR acute renal failure).tw |
|  | #4. (acute kidney insufficie* OR acute renal insufficie*).tw |
|  | #5. Acute tubular necrosis .tw |
|  | #6. (AKI OR ARI OR ARF OR AKF OR ATN) .tw |
|  | #7. OR/1-6 |
|  | #8. exp patient care bundle*/ |
|  | #9. exp care bundle*/ |
|  | #10. care checklist OR care package OR care pathway OR care intervention |
|  | #11. prevention bundle* OR intervention bundle* |
|  | #12. evidence-based intervention* |
|  | #13. quality improv* |
|  | #14. guideline adherence |
|  | #15. OR/8-14 |
|  | #16. 7 and 15 |
| Embase | #1. exp ‘acute kidney failure’/ |
|  | #2. exp ‘kidney failure’/ |
|  | #3. ‘acute kidney injur*’OR ‘acute renal injur*’ |
|  | #4. acute renal failure |
|  | #5. acute kidney insufficie* OR acute renal insufficie* |
|  | #6. Acute tubular necrosis |
|  | #7. acute kidney tubule necrosis |
|  | #8. AKI OR ARI OR ARF OR AKF OR ATN |
|  | #9.or/1-8 |
|  | #10. exp ‘care bundle’/ |
|  | #11. care AND bundle* |
|  | #12. ‘care checklist’ OR ‘care package’ OR ‘care pathway’ OR ‘care intervention’ |
|  | #13. ‘prevention bundle*’ OR ‘intervention bundle*’ |
|  | #14. ‘evidence-based intervention*’ |
|  | #15. 'protocol compliance' OR 'guideline adherence' |
|  | #16. or/10-15 |
|  | #17. #9 AND #16 |
| Cochrane Library | #1. Mesh descriptor [ acute kidney injury ] explode all trees |
|  | #2. (acute kidney injur*OR acute renal injur*). ti, ab,kw |
|  | #3. (acute kidney failure OR acute renal failure). ti, ab,kw |
|  | #4. (acute kidney insufficie* OR acute renal insufficie*). ti, ab,kw |
|  | #5. (Acute tubular necrosis). ti, ab,kw |
|  | #6. (acute kidney tubular necrosis). ti, ab,kw |
|  | #7. (AKI OR ARI OR ARF OR AKF OR ATN) .tw |
|  | #8. Mesh descriptor [renal insufficiency] explode all trees |
|  | #9. or/1-8 |
|  | #10. Mesh descriptor [patient care bundles] explode all trees |
|  | #11. Mesh descriptor [guideline adherence] explode all trees |
|  | #12. (care bundle*). ti, ab,kw |
|  | #13. (care checklist OR care package OR care pathway OR care intervention). ti, ab,kw |
|  | #14. (prevention bundle* OR intervention bundle*). ti, ab,kw |
|  | #15. (evidence-based intervention*). ti, ab,kw |
|  | #16. (quality improv*). ti, ab,kw |
|  | #17. OR/10-16 |
|  | #18. #9 AND #17 |
| Wanfang Data, China National Knowledge Infrastructure | Search words included acute kidney injury, therapy bundle, prospective observational study, uncontrolled before-after study and randomized controlled trial |
